# Attitudes toward working in rural areas of Thai medical, dental and pharmacy new graduates in 2012: a cross-sectional survey

**DOI:** 10.1186/1478-4491-11-53

**Published:** 2013-10-23

**Authors:** Noppakun Thammatacharee, Rapeepong Suphanchaimat, Thunthita Wisaijohn, Supon Limwattananon, Weerasak Putthasri

**Affiliations:** 1 Health Insurance System Research Office (HISRO), Nonthaburi, Thailand; 2 International Health Policy Program (IHPP), Ministry of Public Health, Nonthaburi, Thailand; 3 Banphai Hospital, Khon Kaen, Thailand; 4 Faculty of Pharmaceutical Sciences, Khon Kaen University, Khon Kaen, Thailand; 5 IHPP, Ministry of Public Health, Nonthaburi 11000, Thailand

**Keywords:** Medical graduate, Dental graduate, Pharmacy graduate, Medical education, Supply and distribution, Attitude of health personnel, Rural distribution

## Abstract

**Background:**

Inequity in health workforce distribution has been a national concern of the Thai health service for decades. The government has launched various policies to increase the distribution of health workforces to rural areas. However, little is known regarding the attitudes of health workers and the factors influencing their decision to work in rural areas. This study aimed to explore the current attitudes of new medical, dental and pharmacy graduates as well as determine the linkage between their characteristics and the preference for working in rural areas.

**Methods:**

A cross-sectional survey was conducted, using self-administered questionnaires, with a total of 1,225 medical, dental and pharmacy graduates. They were participants of the meeting arranged by the Ministry of Public Health (MOPH) on 1–2 April 2012. Descriptive statistics using mean and percentage, and inferential statistics using logistic regression with marginal effects, were applied for data analysis.

**Results:**

There were 754 doctors (44.4%), 203 dentists (42.6%) and 268 pharmacists (83.8%) enrolled in the survey. Graduates from all professions had positive views towards working in rural areas. Approximately 22% of doctors, 31% of dentists and 52% of pharmacists selected ‘close proximity to hometown’ as the most important reason for workplace selection. The multivariable analysis showed a variation in attributes associated with the tendency to work in rural areas across professions. In case of doctors, special track graduates had a 10% higher tendency to prefer rural work than those recruited through the national entrance examination.

**Conclusions:**

The majority of graduates chose to work in community hospitals, and attitudes towards rural work were quite positive. In-depth analysis found that factors influencing their choice varied between professions. Special track recruitment positively influenced the selection of rural workplaces among new doctors attending the MOPH annual meeting for workplace selection. This policy innovation should be applied to dentists and pharmacists as well. However, implementing a single policy without supporting strategies, or failing to consider different characteristics between professions, might not be effective. Future study of attitudes and factors contributing to the selection of, and retention in, rural service of both new graduates and in-service professionals was recommended.

## Background

Health workers are one of the six key components of a health system [1,2]. Empirical evidence demonstrates that ensuring the adequate distribution of skilled medical staff results in the improvement of patients’ health outcomes [3,4]. Having sufficient quality and quantity of doctors is thus vital to make health systems function effectively and to ensure the health of the entire population. Other professions are equally important. According to the World Health Organization (WHO), a health workforce refers to ‘all people whose primary intention is to enhance health’; this includes clinical staff such as physicians, nurses, dentists and pharmacists, as well as those who support or manage the system but are not directly involved with providing health services [1].

For more than four decades, Thailand has invested great efforts aimed at increasing the number of health workers through a range of policies [5-8]. This was underpinned by a huge investment in the infrastructure of health facilities in the 1970s which provided coverage for the whole population through the provision of community hospitals in all districts and health centres in all sub-districts [9]. A policy of compulsory rural service through contract bonding was then launched and enforced upon medical graduates, starting in 1972. Graduates had to work for at least 3 years in public facilities under the Ministry of Public Health (MOPH). If they wished to breach their contract, they had to pay a fine of US$1,330 [5]. This contract system was then expanded to other professions such as nurses and dentists; however, working in public facilities remained voluntary for pharmacists. More recently, the past two decades have seen the founding of a number of new health professional schools, mostly outside Bangkok and the vicinity [5,6]. Civil servant posts and financial incentives have been provided, on top of the regular salary, to offset the opportunity loss of working in remote areas [5,8].

In addition, strategies aiming to recruit students from rural backgrounds, alongside promoting exposure of medical trainees to rural work, have been initiated. This corresponds to the guidelines recommended by WHO in 2010 and also various international reports and studies [10-15]. The most clearly targeted programmes are the ‘Collaborative Project to Increase Production of Rural Doctors (CPIRD)’, launched in 1995, and ‘One District One Doctor (ODOD)’ which was launched in 2005 [6,8,16,17]. These provide a special admission mode (special track) in parallel to the existing methods of national entrance examination and direct admission. When seeking to become a doctor through the national entrance examination, any 12th-grade student can apply to sit the exam. Aside from the national entrance exam, some schools also arrange their own recruitment, so-called direct admission, selecting students through their own institution specific exams. For the special track admission, any 12th-grade student residing in one of a number of prescribed provinces which have a shortage of doctors are eligible to sit the exam. Unlike the national entrance exam where competition is at the national-level, applicants for CPIRD have to compete with others within the same province, while ODOD applicants must compete with students from the same district. CPIRD and ODOD students spend the first 3 years of their study in universities alongside students recruited through the normal track, but they spend their 3 clinical years training in accredited regional and provincial hospitals of the MOPH. These hospitals are affiliated with relevant university faculties of medicine and their diplomas are granted by the university, not the MOPH institutes. Graduates from all tracks are obliged to undertake mandatory service in public facilities, 3 years for those recruited through the normal track or CPIRD, and 12 years for those selected through the ODOD scheme. The different recruitment tracks result in different choices of workplace being available upon graduation: students recruited through the CPIRD/ODOD track are required to work in provinces which are experiencing a severe shortage of health workers, within, or near their hometown area. The choice of workplace for normal track graduates is more flexible: the graduates are able to choose from the full range of vacant MOPH posts.

Through all the policies mentioned above, the production of health workers in Thailand increased considerably [7], making the density of doctors plus nurses and midwives per population well beyond the 2006 WHO benchmark of 23 per 10,000 population [18].

Although the production of health workers has grown continuously, Thailand still suffers from the mal-distribution of human resources for health, both between different geographical areas, and between the public and private sectors [19]. Health professionals are highly concentrated in urban areas which provide the greatest opportunities to work in private facilities. This is most clearly seen in Bangkok where recent data showed that the ratio of population per doctor was lowest: around six times lower than that found in the Northeast, the region that has experienced the most critical shortages. This disparity was even more marked in dentists, the difference in the density of dentists to population between Bangkok and the Northeast was around 15 times in 2009. By contrast it was less significant in pharmacists where the difference was much lower (see Table 1) [20].

**Table 1:** Ratio of population per one doctor, dentist and pharmacist in different regions of Thailand

**Region**	**Doctor**^**a**^	**Dentist**^**b**^	**Pharmacist**^**c**^
Bangkok	850 : 1	1,167 : 1	3,667 : 1
Central (excluding Bangkok)	2,683 : 1	8,945 : 1	7,609 : 1
North	3,279 : 1	9,858 : 1	7,728 : 1
South	3,354 : 1	10,143 : 1	7,598 : 1
Northeast	5,308 : 1	17,663 : 1	11,171 : 1

Source: Human Resources for Health Research and Development Office, Thailand.

^a^Data in 2007.

^b^Data in 2009.

^c^Data in 2012.

The internal brain drain from the public sector to the private sector has been gradual, but has grown since 2000: this has been caused by the promotion of international trade in health services and Thailand’s economic recovery from the 1997 Asian financial crisis [8]. This situation became more complex with the introduction in 2001 of the Universal Coverage Scheme (UCS). Roles of health staff have been changed with greater emphasis now placed on prevention and promotion health services rather than solely on curative care [21]. This has increased the workloads of health workers because the removal of the financial barrier to care has stimulated an increased demand for services from patients [22]. A large number of health professionals have left public health facilities to join the private sector [23]. Recent data from the MOPH illustrated that the number of doctors working full-time in private hospitals has risen by around 20% between 2000 and 2010 [20]. In 2009 dentists serving in the private sector constituted about 7.2% of the entire dentist population; this is almost double the figure in 1971 when it stood at 3.8% [24].

With the background mentioned above, this study set out to investigate the attitudes of graduates towards working in rural areas, and the factors which are linked with the preference to work in these areas. Though this is a critical issue, there were few domestic studies exploring this problem and most studies confined their scope to just doctors [25]. This study aimed to fill this knowledge gap and examine three of the main health professions: doctors, dentists and pharmacists. It is hoped that the findings will be useful in ensuring that current and future policy is more responsive to Thailand’s health workforce problems.

## Methods

### Study design and target population

A cross-sectional survey was performed on 1 and 2 of April 2012 in Thailand’s annual health worker meeting which is jointly arranged each year by the MOPH and the health professional associations. The meeting aims to orientate new graduates about choices of workplace, career path and opportunities for future study. The MOPH also uses this occasion for the compulsory selection of MOPH workplace for new graduates from among the vacant posts available. There were 1,697 new medical, 476 dental and 320 pharmacist graduates participating in the meeting. During the meeting graduates select the posts they would like to take up in a series of rounds. They go to a booth representing the province they wish to work in and register for a post. In the event of oversubscription in the area in which they wish to work, graduates must draw lots to select the successful candidates. Those who are unsuccessful in the drawing of lots must enter the following round to find a post in one of the provinces with unfilled positions.

As taking an MOPH post is obligatory for all doctors and dentists, the 1,697 new medical and 476 dental graduates present made up the majority of the whole batch of graduates from 2012 in Thailand. Taking an MOPH post is voluntary for pharmacists. Nurses do not participate in this meeting because they choose their workplace at the time of their enrolment in nursing school. The survey was part of a routine monitoring of graduates, conducted annually by the International Health Policy Programme (IHPP), under the MOPH, since 2010. In the first two years, pharmacists were not enrolled in the survey. Year 2012 was the first time in which the comprehensive survey of all the three professions was performed. The main content and objectives of the questionnaire remained unchanged since 2010, however, some text was amended to ensure concision and clarity.

### Survey tool

A self-administered, structured, close-ended questionnaire was used. It was composed of three sections: (1) respondents’ general characteristics/attributes; (2) workplace preference and factors influencing their choice; and (3) attitudes towards rural practice. The demographic questions in section 1 asked graduates to specify their age, sex, location of hometown or places they were brought up (Urban *versus* Rural), the location of their professional school (Bangkok and vicinity *versus* Upcountry), mode of admission (National entrance examination *versus* Direct admission *versus* Others, for example, special quota including CPIRD/ODOD), parents’ education (Below bachelor degree *versus* At least bachelor degree) and parents’ career (Civil servant *versus* Not civil servant). It should be noted that ‘Urban’ in this study was defined as Bangkok and the vicinity (Bangkok and four surrounding provinces: Nonthaburi, Pathumthani, SamutPrakarn and SamutSakorn) and the provincial city (Central district) of other provinces, while ‘Rural’ referred to any village, town or non-provincial city.

In section 2, respondents were asked to choose where they would prefer to work if they had complete freedom to choose and were not bound to complete mandatory public service. The choices of workplace provided in this question were ‘community hospitals’ , ‘provincial hospitals’ and ‘others such as private hospitals or continuing higher education’. They were then asked to indicate only one factor, from the list shown in the questionnaire, which had the greatest influence on their choice. These factors included the ‘Close proximity of hometown’ , ‘High income’ , and ‘Good support from colleagues’ , etc.

In the last section, attitudes towards rural work were measured using the standard Likert scale indicating whether they agreed with six opinions shown in the questionnaire. The six opinions comprised of four positive statements and two negative statements as follows: (1) ‘Rural colleagues are nice and friendly’; (2) ‘Rural colleagues are helpful’; (3) ‘Rural work opens chances to utilize different medical skills’; (4) ‘Rural work is challenging’; (5) ‘There are very few facilities in rural areas’; and (6) ‘Working in rural areas means being separated from family and friends’. The ranks ranged from 1 ‘Strongly disagree’ to 5 ‘Strongly agree’.

The questionnaire was distributed to the new graduates after the workplace selection process had been completed. On average each questionnaire took between 5 and 10 minutes to complete. A validity check was performed through a consultative meeting among three experts in Thailand’s MOPH. A pilot test of the questions on rural attitudes was conducted, to test their reliability, using final-year medical students in KhonKaen University before the actual survey commenced (Cronbach’s alpha = 0.64 - Considered acceptable reliability).

### Data analysis

STATA software Version 11 (STATA Corporation, College Station, TX, USA) was used for data analysis. The analysis consisted of three parts: first, descriptive statistics were used to describe the demographic profiles of graduates and general findings of workplace selection, factors influencing their choice and rural attitudes. Second, univariable analysis was performed, using Pearson’s Chi-square test, to determine an association between individual attributes (independent variables) and the choice of workplace (dependent variables); in this case, dependent variables were coded either ‘rural preference’ , if respondents chose to work in community hospitals, or ‘urban preference’, if they selected other options. Finally, multivariable analysis was conducted to find out the relationship between individual attributes and rural preference, taking into account all potential confounders. The attributes selected in the model were those yielding statistical significance over 95% level of confidence in the univariable analysis. Attributes which were recommended by WHO guidelines [10] were also recruited into the model, for instance, ‘location of the school outside Bangkok and vicinity’ and ‘rural hometown’. The results presented an absolute probability rather than a relative tendency as is commonly shown in odds ratios.

## Results

A total of 1,225 graduates participated in the survey. They were 754 doctors, 203 dentists and 268 pharmacists, corresponding to a response rate of 44.4%, 42.6% and 83.8%, respectively.

### General characteristics of graduates

The mean age of graduates was approximately 24 years and women made up the majority of participating graduates in all professions. Over three-quarters of medical and dental graduates were brought up in urban areas while around half of the pharmacists had their hometown in a rural area. Most medical and dental graduates had graduated from universities located in Bangkok and the vicinity, whereas almost all the pharmacists were from pharmacy schools in upcountry areas. The national entrance examination and direct admission were the main routes of admission in all professions, but a significant minority of medical graduates (22%) were recruited through the CPIRD/ODODprogrammes. Around two-thirds of all graduates had parents with at least a bachelor’s degree. A slight difference in the career of graduates’ parents across professions was observed. The parents of around half the medical and dentistry graduates held a civil service post, this contrasts with 60% of the parents of pharmacy graduates (see Table 2).

**Table 2:** General characteristics of graduates in each profession

**Attribute**	**Doctor (%)**	**Dentist (%)**	**Pharmacist (%)**
	**<** ***n *** **= 754>**	**<** ***n *** **= 203>**	**<** ***n *** **= 268>**
Mean age in years (SD)	24.1 (0.9)	24.3 (1.6)	23.9 (1.6)
Sex			
● Male	295 (39.5)	61 (30.0)	57 (21.3)
● Female	452 (60.5)	142 (70.0)	120 (78.7)
Hometown area			
● Urban	568 (75.9)	157 (77.7)	127 (47.7)
● Rural	180 (24.1)	45 (22.3)	139 (52.3)
School location			
● Bangkok and vicinity	411 (57.3)	136 (67.0)	9 (3.4)
● Upcountry	306 (42.7)	67 (33.0)	258 (96.6)
Mode of admission			
● National entrance examination	200 (26.6)	85 (41.9)	159 (59.6)
● Direct admission	313 (41.6)	89 (43.8)	70 (26.2)
● CPIRD/ODOD	167 (22.2)	NA	NA
● Others (for example, special quota)	72 (9.6)	29 (14.3)	38 (14.2)
Parents’ education			
● At least bachelor degree	539 (72.1)	135 (66.8)	163 (61.1)
● Below bachelor degree	209 (27.9)	67 (33.2)	104 (38.9)
Parents’ career			
● Civil servant	387 (51.3)	100 (49.3)	153 (59.1)
● Not civil servant	377 (48.7)	103 (50.7)	106 (40.9)

^a^*n*= total number of respondents; NA = not applicable as dentists and pharmacists did not have CPIRD/ODOD admission programme; SD = standard deviation.

### Workplace preference and reasons influencing workplace selection

**Figure 1:**
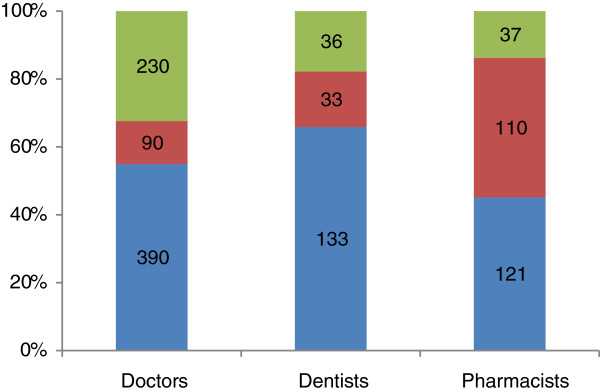
**Workplace preference given having freedom to choose workplaces.** Square blue box: community hospitals, square red box: provincial hospitals, square green box: others.

Under the assumption that they were not compelled by the bond to work in an MOPH facility, more than half of the medical and dental graduates said they would choose to work in community hospitals. Around one-third of doctors selected other workplaces outside the public sector or wished to continue further education. ‘Community hospitals’ was also the choice most commonly selected by pharmacist graduates, slightly more popular than provincial hospitals; fewer than 15% of pharmacists wished to work outside the public sector (see Figure 1).

Of the reasons influencing workplace selection, ‘Close proximity to hometown’ was of the greatest importance for all professions. Approximately 22% of doctors, 31% of dentists and 52% of pharmacists selected it in first place. Other important factors for dentists and pharmacists were quite similar. These two groups showed marked concern for ‘High income’ (22% of dentists and 14% of pharmacists selecting this factor) and ‘Desired type of work’ (15% of pharmacists and 9% of dentists selecting this factor). By contrast, doctors were less motivated by these reasons: they tended to select workplaces where they were able to seek good support from colleagues and gain new working experiences (see Table 3).

**Table 3:** Proportion (%) of graduates selecting each reason as the greatest importance to the total graduates

**List of reasons**^**a**^	**Doctor**	**Dentist**	**Pharmacist**
Close proximity of hometown	21.6	30.5	52.2
High income	5.7	22.1	14.2
Good support from colleagues	16.5	NA	NA
Gaining new experience	14.7	6.4	6.0
Well-known workplace	NA^†^	6.9	4.0
Desired type of work	NA^†^	8.9	14.6
High chance to pursue specialty training in the future	6.9	3.5	0.4
Appropriate workload	5.3	0.5	<0.1
Good environment	1.3	19.2	4.9
Having friend(s) to go with	11.4	NA^2^	NA^2^

^a^Only key reasons from the whole list of reasons in the questionnaire are shown.

NA = Not applicable as that reason did not present in the questionnaire for particular profession.

### Attitudes towards rural work

**Figure 2:**
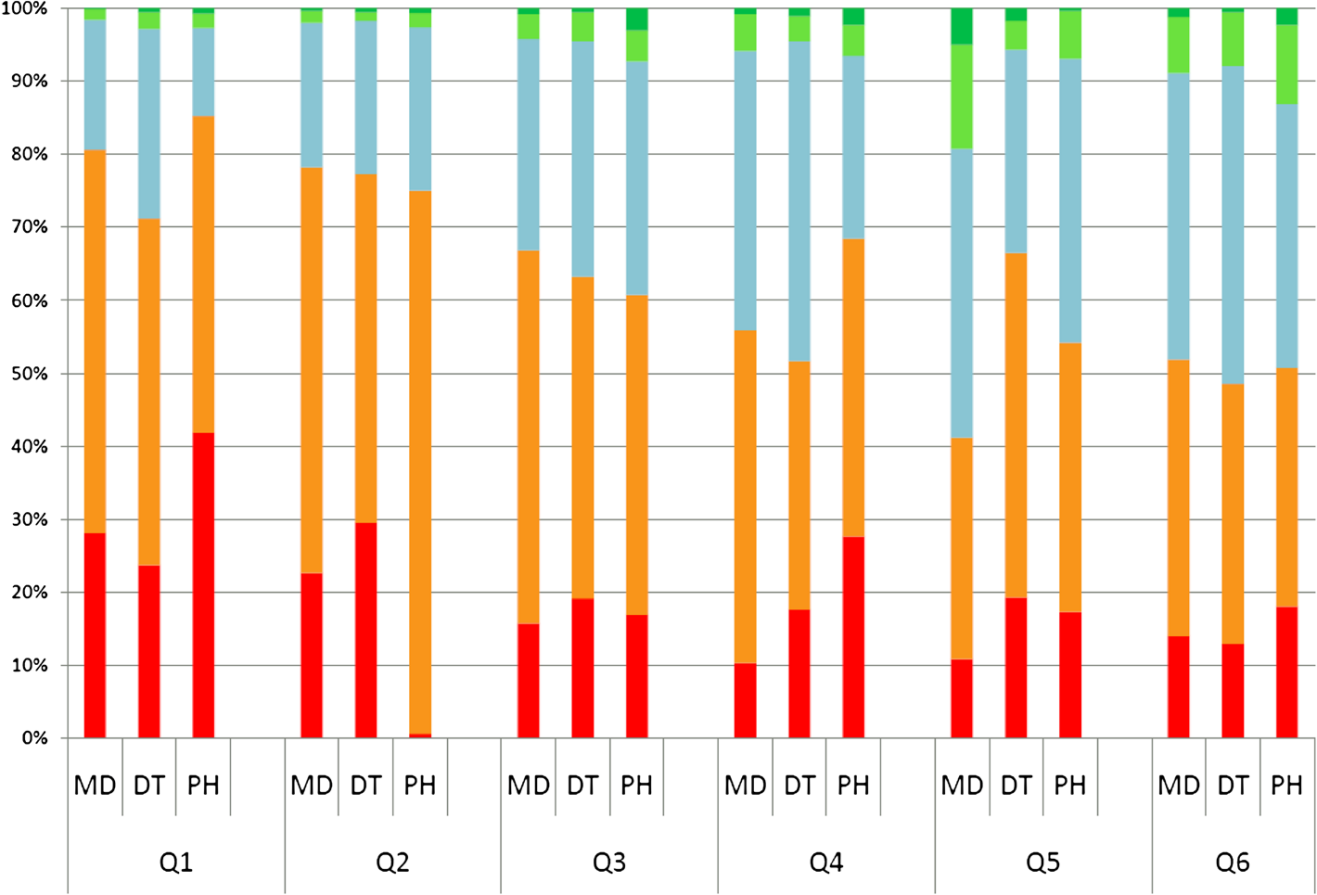
**Attitudes towards rural work.** Square red box:strongly agree, square yellow box:agree, square gray box:neutral, square yellow green box:disagree, square green box:strongly disagree. Note: Q1 = Rural colleagues are nice and friendly. Q2 = Rural colleagues are helpful. Q3 = Rural work opens chances to utilize different medical skills. Q4 = Rural work is challenging. Q5 = There are very limited facilities in rural areas. Q6 = Working in rural areas means being separated from family and friends.

Overall, there were slight differences in the agreement on each view between professional groups. The highest level of agreement came in response to the first two views, ‘Rural colleagues are nice and friendly (Q1)’ and ‘Rural colleagues are helpful (Q2)’: around 70% to 80% of all respondents chose positive answers on these attitudes. However, the proportion of pharmacists expressing ‘strongly agree’ on Q2 is much lower than that of other professions. The next highest level of agreement were found in response to the statement ‘Rural work opens chances to utilize different medical skills (Q3)’ and ‘Rural work is challenging (Q4)’. On the negative opinions: ‘There are very limited facilities in rural areas (Q5)’ and ‘Working in rural areas means being separated from family and friends (Q6)’ , around half of all graduates stayed neutral or disagreed. The proportion of dentists stating ‘strongly agree’ or ‘agree’ on Q5 was slightly higher than that of the other two groups (see Figure 2).

### Association between preference for rural work and the attributes of graduates

Univariable analysis suggested that, for medical graduates, the mode of admission was significantly associated with the preference for rural work. Those admitted through CPIRD/ODOD tended to choose community hospitals as their preferred workplace. Of 167 CPIRD/ODOD graduates, 98 doctors showed a preference for rural work, constituting 25% of those selecting community hospitals. The relationship with hometown area was of borderline significance in the cases of dentist and pharmacist. Thirty-five of 45 dental graduates, and 71 of 139 pharmacist graduates, who spent their childhood in rural areas, preferred to work in rural areas (see Table 4).

**Table 4:** Association between rural preference and graduates’ attributes: a univariable analysis

**Graduates’ attributes**	**Having rural preference (serving community hospitals)**		
	**Doctor (%)**	**Dentist (%)**	**Pharmacist (%)**
	**< *n *** **= 390>**	**< *n *** **= 133>**	**< *n *** **= 121>**
Sex	*P*value = 0.577	*P*value = 0.553	*P*value = 0.803
● Male	154 (39.8)	42 (31.6)	25 (20.7)
● Female	233 (60.2)	91 (68.4)	96 (79.3)
Hometown area	*P*value = 0.205	*P*value = 0.052	*P*value = 0.055
● Urban	289 (74.9)	97 (73.5)	50 (41.3)
● Rural	97 (25.1)	35 (26.5)	71 (58.7)
School location	*P*value = 0.250	*P*value = 0.151	*P*value = 0.957
● Bangkok and vicinity	220 (59.6)	85 (63.9)	4 (3.3)
● Upcountry	149 (40.4)	48 (36.1)	117 (96.7)
Mode of admission	*P*value = 0.044^a^	*P*value = 0.466	*P*value = 0.147
● National entrance examination	94 (24.2)	60 (45.1)	79 (65.3)
● Direct admission	165 (42.4)	55 (41.4)	25 (20.7)
● CPIRD/ODOD	98 (25.2)	NA	NA
● Others (for example, special quota)	32 (8.2)	18 (13.5)	17 (14.0)
Parents’ education	*P*value = 0.658	*P*value = 0.529	*P*value = 0.751
● At least bachelor degree	279 (72.1)	90 (68.2)	72 (60.0)
● Below bachelor degree	108 (27.9)	42 (31.8)	48 (40.0)
Parents’ career	*P*value = 0.172	*P*value = 0.084	*P*value = 0.708
● Civil servant	192 (49.2)	71 (53.4)	70 (60.3)
● Not civil servant	198 (50.8)	62 (46.6)	46 (39.7)

^a^Statistical significance over 95% level of confidence.

*n*= total number of graduates choosing ‘Community hospitals’ in the questionnaire, NA = not applicable as dentists and pharmacists did not have CPIRD/ODOD admission programme.

According to the selection criteria mentioned in the Methods section above, ‘mode of admission’ was thus selected for further analysis, using multivariable logistic regression with the marginal effect model. School location and hometown area were selected in the model too, despite having no statistical significance over 95% level of confidence. This was because the two variables matched the recommendations set by WHO: ‘A1-Students from rural background’ and ‘A2-Health professional schools outside major cities’ [10].

Table 5 demonstrates the full results of multivariable analysis with marginal effect. CPIRD/ODOD doctors had a 10% higher probability of preferring rural work than graduates recruited through the national entrance exam path, with a borderline significance (*P*value = 0.058). Location of hometown and placement of medical school were not significantly linked with the preference for rural work. For dentists and pharmacists, there were no attributes significantly influencing the intention to work in rural areas. However, when relaxing the interpretation of confidence level to 10% (*P*<0.10), dentists whose childhood was spent in rural areas tended to have a positive preference for rural work, 15% more than those with an urban background. The same pattern was observed in pharmacists where having a rural hometown resulted in a 9% higher tendency in choosing to work in rural areas (see Table 5).

**Table 5:** Association between rural preference and graduates’ attributes: a multivariable analysis with marginal effect

	**Doctor**			**Dentist**			**Pharmacist**		
	**Coef.(SE.)**	**[95% CI]**	***P*** **value**	**Coef.(SE.)**	**[95% CI]**	***P*** **value**	**Coef.(SE.)**	**[95% CI]**	***P*** **value**
Rural hometown^a^	0.05 (0.05)	[−0.05 0.14]	0.322	0.15 (0.09)	[−0.01 0.32]	0.071	0.09 (0.06)	[−0.02 0.21]	0.114
School in upcountry^b^	−0.07 (0.04)	[−0.15 0.08]	0.079	0.09 (0.07)	[−0.05 0.23]	0.214	0.00 (0.17)	[−0.33 0.33]	0.988
Mode of admission^c^									
● Direct admission	−0.05 (0.05)	[−0.14 0.04]	0.257	−0.06 (0.07)	[−0.20 0.08]	0.376	−0.11 (0.07)	[−0.25 0.03]	0.137
● CPIRD/ODOD	0.10 (0.05)	[−0.00 0.21]	0.058	NA	NA	NA	NA	NA	NA
● Others	−0.03 (0.07)	[−0.16 0.11]	0.722	−0.08 (0.10)	[−0.05 0.23]	0.464	−0.05 (0.09)	[−0.23 0.12]	0.573

^a^Having urban hometown is a comparator.

^b^Graduating from school within Bangkok and vicinity is a comparator.

^c^Being admitted through the national entrance examination is a comparator.

95% CI = 95% confidence interval; Coef.(SE.) = Coefficient (standard error) - the coefficient represented absolute probability (%) of having rural preference in each attribute compared to base attribute (comparator). NA = Not applicable as dentists and pharmacists did not have CPIRD/ODOD admission programme.

## Discussion

The majority of graduates preferred to work in community hospitals, and attitudes towards rural work were positive or at least neutral in most cases. Nonetheless, in-depth analysis found that the factors influencing their choice varied across professions. Unlike ‘Close proximity to hometown’ , which was the most important factor consistent in every profession, high income was a key deciding factor for dentists and pharmacists but not for doctors. New medical graduates paid more attention to the opportunity to acquire more experience, and to get support from colleagues. This finding does not necessarily mean that doctors place a lower priority on financial rewards. Instead, it might implicitly reflect the financial imbalances in Thailand’s health system, and the way doctors are paid. Rural work is not a barrier to high income for doctors as they are still able to earn more than dentists and pharmacists. New medical graduates, working in rural areas, automatically gain additional income, on top of their regular salary, offsetting their opportunity loss if they choose rural work. This amounts to US$1,000/month in the first 3 years, it then increases to >US$2,000/month if they serve in rural areas for up to 10 years. Dental graduates are also entitled to this additional income but pharmacists are not. Dentists and pharmacists are unable to match doctors in boosting their earnings through performing medical procedures outside official working hours. For example, a doctor can earn US$13 per case of shock/heart failure treatment, and US$27 per appendectomy [26,27]. Thus the revenue doctors can earn does not differ markedly whether they work in rural or urban MOPH health facilities. This may explain why the workplace selection of doctors depended more on other reasons.

The attribute that showed the strongest association with the choice to work in a rural area was having received training following entry on the CPIRD/ODOD admission programme, this had borderline statistical significance. This was understandable as the programme itself represented a bundle of policies, and was unique in terms of admission criteria, curricular design and workplace selection. Graduates from this track were obliged to serve a community in an area with a pre-existing shortage of doctors, either in or near their hometown. Having been trained in MOPH affiliated hospitals during their clinical years, they might feel more confident in performing clinical work in the real setting. This, in turn, may make them feel familiar with real workplaces, confident in handling the type of patients commonly found in MOPH health facilities, and able to seek professional peer support in the future [28]. This explanation of the association between special track recruitment and the choice to work in a rural area corresponded to a number of previously published studies [11,29-33].

It was interesting that, independent from the effect of other factors, merely having a rural background did not strongly determine the preference of dentists and pharmacists to work in rural areas. However, a positive link between rural background and intention to undertake rural service was found and this might be an important indicator. During the policy formulation process this point should not be ignored; however, it might not be effective in addressing health workforce maldistribution unless various supporting mechanisms are also in place.

Policy-makers may benefit from the evidence shown in this study. A special track programme like CPIRD/ODOD could potentially help increase the number of dentists and pharmacists serving rural communities. However, policy implementation is not a one-size-fits-all process. It is important to consider the difference between professions in the attitudes towards rural work and the factors attracting graduates to work in rural areas. A bundle of supporting mechanisms, such as promoting supportive work environments and enhancing financial incentives for dentists and pharmacists, should be set up in parallel.

Despite rigorous design and analysis, this study still faced some limitations. Graduates enrolled in the survey could not represent all the students graduating in 2012. This was because around 20% of total graduates did not attend the MOPH selection meeting: the missing 20% included those who opted out from mandatory service by paying the fine in order to enter private practice. These included graduates who chose to work in non-MOPH public facilities, for example, military service, or continue specialty training in some areas allowed by the Medical Council of Thailand, such as forensics or pathology. A selection and information bias might have occurred and this could affect the precision of the results. Assuming that all new medical graduates not attending the annual meeting were willing to pay the fine to avoid rural placement, a positive correlation between the various factors and the preference to work in rural areas would be overestimated as the dataset of this survey confined only in students who conformed to the rural mandatory services imposed by the MOPH. This is also another point of limitation. When extrapolating the results of this study to the whole health professional graduates, the degree of this correlation will probably be diluted. One potential solution to avoid this bias is conducting a survey on students who are about to leave their professional schools in order to gain insights from students who plan to opt out from the contract bound with the MOPH, which is a plan for future studies.

Despite the fact that the result of this study might be affected by the non-response bias, the positive correlation of special track recruitment with rural preference is likely to persist and still valid in the actual practice. This was confirmed by, and consistent with, findings from other domestic literatures [25,34], for instance, Pagaiya et al. explored the secondary data of medical graduate registry between 2001 and 2007 and found that the median survival years in the rural area of special track graduates were in the range of 4 to 10 years, significantly higher than the median survival years of 3 to 6.5 years in normal track graduates. This was equivalent to the 12% lower risk of leaving rural areas in special track graduates in contrast to that of normal track graduates (hazard ratio = 0.88, *P*value = 0.001) [35].

The nature of respondents was also inhomogeneous; participation in this meeting is voluntary for pharmacists while doctors and dentists are compelled to attend. Pharmacists, joining and participating in this ceremony might be driven by other motivations. For instance, they may wish to gain a civil service post, and thus decide to join this meeting even if they are not motivated to work in a rural area. They might participate in this meeting in order to gain insights about their potential career path and opportunities for their future, rather than as a result of a direct intention to sign a contract to work in the public sector.

One important consideration is the limitations of the design and data collection. As this study is a cross-sectional survey, the results shown here were just a static picture which could not capture changes or trends in job preferences and factors affecting workplace selection. This is due to the fact that although the survey has been conducted regularly, 2012 was the first year that all three professions were enrolled in the survey. In the future, presuming the survey continues to be conducted annually, it will be possible to examine the trends and changes in the attitudes of graduates in all three professions. However, comparisons of the results between professions must be conducted with caution. This is because of the selection bias mentioned earlier and the variations in the questionnaire between the respondent groups, in particular the questions regarding the reasons influencing workplace selection. The model of analysis presented here intended to determine the association of various factors with the intention to work in rural areas, it did not seek to predict the likelihood of graduates choosing to work in rural areas. This would require a more deliberate model design.

Last but not least, the results of this survey were a subjective assessment. This may not reflect the real ability to retain health workers in rural areas. Workforce retention depends not only on the intention of the individuals involved, but also on other health system components and the broader contextual environment. To obtain a better understanding, a qualitative study complementing this quantitative survey is recommended. Additional studies of new health professional graduates alongside those who are in service are suggested.

## Conclusion

The majority of graduates chose to work in community hospitals, and attitudes towards rural work were quite positive across all the selected professions. Nonetheless, in-depth analysis found that the factors influencing their choice varied between the professions. Special track recruitment positively influenced selection of a rural workplace amongst new doctors. Special track recruitment positively influenced the selection of rural workplaces amongst new doctors attending the MOPH annual meeting for workplace selection. This policy innovation should be applied to dentists and pharmacists as well. However, implementing a single policy without other supporting strategies might not be effective in addressing health workforce shortages. A bundle of policies such as, recruiting more students from rural areas, setting up special admission/curriculum programmes which allow early rural exposure, and leveraging financial incentives should be promoted. Differences in the attitudes and characteristics of graduates which affect their choice to work in rural areas should be considered during policy formulation and implementation. Future study of the attitudes and factors contributing to the selection of, and retention in, rural service of both new graduates and in-service professionals is recommended.

### Ethics approval

While informed consent was sought and protection of confidentiality was strictly followed; the National Ethical Review Committee waived ethical clearance as this is a regular monitoring work by the Government as shown in the letter Ref IHRP 47.2/2553 date 28 January 2553 BE (2010 AD).

## Competing interests

The authors declare that they have no competing interests.

## Authors’ contributions

NT, TW, RS and WP designed the study and contributed to data collection. NT, RS and SL were responsible for data analysis. All authors contributed to the shaping of this manuscript and approved the final version.
